# Theory of mind and executive functions in sighted children of blind parents

**DOI:** 10.3389/fpsyg.2025.1715624

**Published:** 2026-02-09

**Authors:** Joanna Wysocka, Maciej Haman, Karolina Golec, Agnieszka Pluta

**Affiliations:** Faculty of Psychology, University of Warsaw, Warsaw, Poland

**Keywords:** blind and visually impaired people, executive functions, false belief task, social development, Theory of Mind

## Abstract

**Introduction:**

Previous research indicates that during infancy, children of blind parents demonstrate greater communicative flexibility compared to peers raised by sighted parents. The present study aimed to examine whether such experience may influence executive functions and theory of mind at later stages of development.

**Methods:**

Children raised by sighted parents as well as children whose parent(s) have a visual impairment participated in the study. They completed tasks related to belief attribution (custom computer task, Theory of Mind Task Battery) as well as a task measuring executive functions (HEI-shift task).

**Results:**

The results were consistent with developmental predictions: in both groups, children performed better on the tasks as they got older. However, no difference between the two groups (children of sighted parents vs parents with visual impairments) was found. Models that included only the participants’ age explained the results better than those that included the group factor.

**Discussion:**

The results suggest that children raised by parents with visual impairments develop the examined skills in a manner typical for their age and comparable to peers raised by sighted parents. Due to the sample size, and heterogeneity of the group, further research is required. Since the study did not indicate significant differences in the developmental trajectory of ToM, parents and caregivers can support the development of ToM in ways already demonstrated effective in other research. For instance, engaging in conversations that include mental state talk can foster the growth of ToM in children.

## Introduction

Theory of Mind (ToM) can be defined as the ability to understand the mental states of others: their beliefs, intentions, preferences, desires. In children, it is typically measured by False Belief Tasks (FBT) (Wimmer and Perner, 1983). There are various FBT versions, one of the most frequently used being the *change of location* paradigm, developed by Wimmer and Perner (1983). In such a scenario, a protagonist places an object (e.g., a toy) in location A and then leaves the scene. Meanwhile, in the protagonist’s absence, the object is moved to location B. Participant’s task is to report where the protagonist believes the object to be, or where the protagonist will look for the object when he/she returns. To answer correctly, participants must understand that the protagonist’s behavior will be driven by their belief about the actual state of affairs and that this belief might not necessarily be consistent with what the participant believes to be true. Numerous studies have demonstrated that typically developing children pass verbal FBT by the age of five, as the accuracy of their answers emerges strikingly in the preschool age (Wellman et al., 2001).

What drives this developmental shift is still a subject of ongoing discussion and research. The authors highlight, among other factors, the role of executive functions (EF) (Zelazo et al., 1997) and language (e.g., Schick et al., 2007). Additionally, studies have emphasized the significant impact of early interactions and the environment on ToM. Investigating the impact of the early social environment on human neurocognitive development is considered crucial, since it enables early intervention for children at risk of adverse outcomes (e.g., Bick and Nelson, 2016). At the same time, it may also contribute to identifying potentially beneficial factors that may boost the development early on (e.g., Kovács and Mehler, 2009).

Substantial insights may be provided by research with participation of sighted children, who are raised by parents who are blind (SCBP, sighted children of blind parents). Although the number of studies in SCBP is strictly limited, they demonstrate that SCBP do not show impairments in social-communication skills in infancy (Senju et al., 2013, 2015; Ganea et al., 2018). Interestingly, they are prone to adapt their communicative behavior to different adult partners, e.g., already at the age of 6 months, they tended to direct their gaze less frequently towards blind parent’s face and, in turn, vocalized more during interaction than did controls towards their sighted parents. However, SCBP did not show the overall reduction of attention to the eyes while watching unfamiliar adults (Senju et al., 2013). This may suggest that already in early infancy, SCBP adjust their communication to the perspective of the interaction partner. Interestingly, they also scored high in the overall developmental measures, in this case, Mullen Scales of Early Learning, mainly in the areas of visual memory and attention (Senju et al., 2013, 2015). Although the studies on gaze processing mechanisms in SCBP reveal mixed results (Senju et al., 2013, 2015), SCBPs’ flexible communicative skills have been confirmed in the most recent study (Ganea et al., 2018). While interacting with their blind parents, SCBP showed fewer communicative actions (e.g., holding something up to show it, pointing) in comparison with the control group of infants interacting with their sighted parents. However, SCBP and control group did not differ in the number of communicative actions while interacting with sighted experimenters. Taken together, the results suggest that SCBP are likely to switch between various communication strategies and adjust them to the person they interact with already in the first year of life. It seems vital to examine how this early flexibility may influence SCBPs’ cognitive and social functioning, especially in terms of ToM.

Given the vital role of visual forms of communication in typical development, one might expect that constrained face-to-face communication with a parent might have an unbeneficial effect on SCBPs’ mentalizing abilities. For instance, a great portion of studies report that early joint attention, defined as a shared focus of two individuals on an object, may scaffold further ToM development (Sodian and Kristen-Antonow, 2015). Also, Baron-Cohen and Cross (1992) considered eye contact (e.g., detecting eye direction, shared attention mechanism) as a base for the later emerging mentalizing abilities. ToM research with participation of blind children showed that they pass ToM tasks with a delay, indicating that visual deprivation may change the trajectory of ToM development (Peterson et al., 2000). However, the above-described studies with participation of SCBP did not document any impairments in their general socio-communicative functioning, quite the contrary - it has been suggested that SCBP seem to employ adaptive, efficient ways of communicating with their blind parents and easily switch between alternative communicative behaviors when they interact with sighted adults (Ganea et al., 2018). It, therefore, appears justified to expect that SCBPs’ early experience might have beneficial rather than adverse effects on their future social outcomes.

The competing theoretical approaches regarding ToM development may result in distinct predictions for the mentalizing abilities in SCBP. According to the *competence change* account, the reason why younger children fail on ToM tasks is because they are not yet able to accurately attribute mental states to the others (Wellman et al., 2001). This *competence change* is likely to occur as a result of gradually acquired experience: while growing old, children encounter various opportunities to reflect upon the difference between their own mental states, those of others, and reality, which facilitates their ToM abilities (Brown et al., 1996). For instance, it is suggested the more children are exposed to talk about thoughts and other unobservable mental states, the higher they score in FBT (Peterson and Slaughter, 2003). Accordingly, deprivation of early conversational experience results in difficulties in ToM understanding observed in deaf children raised by hearing parents, even when controlled for syntax abilities (Woolfe et al., 2002). Being reared by a blind parent, SCBP encounter multiple opportunities to reflect upon the discrepancy between their own and parent’s beliefs, and, as a result, they may come to the earlier realization that visual access serves as a source of knowledge This experience can result in ToM enhancement, however, it may be narrowed to a specific context of classic FBT scenarios, based on *seeing leads to knowing* rule. However, in contrast to the *competence chang*e account, *performance* account posits that children may be able to attribute beliefs before the age of four, but fail to solve verbal FBT because it requires a certain level of cognitive abilities. Such an approach seems to be consistent with hotly discussed findings on spontaneous mental tracking, which has been observed already in the first year of the infant’s life (for the review, see Haman, 2019). Many past studies reported the functional link between executive functions (EF), especially conflict EF and ToM performance, since choosing the proper answer may require preschoolers to inhibit their own prepotent perspective on the situation (Carlson et al., 2015). Moreover, Rubio-Fernández and Geurts (2013) demonstrated that if FBT are implemented with minimized linguistic, attentional, and executive demands, it becomes possible for 3 years old to succeed. Interestingly, there might be specific circumstances, which facilitate both EF and ToM development in young children. McAlister and Peterson (2013) showed that preschool children who had child-aged siblings score higher on both EF and ToM tasks, not only contemporaneously, but also in the long term. One possibility which may account for these results is that various games, frequently played among siblings, require the engagement of EF inhibition skills (e.g., avoiding calling out in hide-and-seek or waiting for a child’s turn in rule-based activities). Other insights into the relationship between early experience and further cognitive and social outcomes comes from research in bilingual children, who outperform their peers in verbal FBT (e.g., Goetz, 2003; Kovács, 2009), most likely due to well-developed inhibitory and selection processes, strengthened by the frequent experience of selecting one, adequate language and inhibit the other (Bialystok and Martin, 2004; Kovács, 2009). Bilinguals’ skills might be to some extent comparable to those observed in SCBP. It might be therefore hypothesized that, similarly to bilingual children, SCBPs’ level of EF might be enhanced as well. If so, in accordance to the *performance* account, SCBPs’ advantage in ToM tasks will not be dependent on certain task properties (e.g. *seeing leads to knowing* scenarios), since their FBT success is more likely to be facilitated by well-developed EF, enabling to tackle multiple aspects of ToM reasoning.

### Summary and study objectives

To date, the unique group of sighted children reared by blind parents has rarely been examined and the only few studies addressing this topic focused entirely on infancy. As a result, whether SCBPs’ early social environment and the observed flexible communicative abilities influence their functioning later in development, remains unknown. Moreover, classic FBTs rely on visual information (the protagonist either sees or does not see the change in the location of the toy). There is a lack of procedures that use other modalities (e.g., auditory), especially when it comes to studying populations with diverse experiences in this area, such as children of parents with visual impairments.

The main objective of this study is to fill this gap and explore whether and how ToM and EF develop in this group compared to their peers reared by sighted parents by answering the following questions: (1). Will the results of SCBP in the area of mentalization and executive functions differ from the results obtained by their peers who are raised by sighted parent(s)? (2). If so, what will be the direction of these differences?

Based on the approach, which states that ToM development is driven by the gradual acquisition of mental-state concepts through experience with conversations about perceptual access and epistemic states (Brown et al., 1996), then SCBP, who frequently encounter discrepancies between their own and their parents’ knowledge states, may show enhanced performance specifically in classic false-belief tasks relying on the “seeing-leads-to-knowing” principle. On that basis, the following hypotheses may be formed: (1). SCBP will outperform controls in visual false-belief tasks, (2). This advantage will be task-specific and may not generalize to non-visual ToM measures.

However, based on the approach that ToM success in preschoolers depends primarily on domain-general processes such as EF (e.g., inhibition, attentional control), as well as considering the previous studies showing that SCBP switch between various communication strategies and adjust them to the person they interact with already in the first year of life, it may be hypothesized that early communicative flexibility observed in SCBP may foster stronger EF, which in turn would facilitate ToM reasoning across contexts. Therefore, (1). SCBP will show enhanced EF performance relative to controls. (2). SCBP will show enhanced ToM performance across both visual and non-visual tasks, with their advantage not restricted to perceptual-access scenarios.

## Materials and methods

### Participants

A total of 42 participants took part in the study. Children of parents with visual impairments were primarily recruited through announcements posted on social media groups dedicated to blind parents. The control group was recruited via announcements published on the laboratory’s social media page.

The SCBP group consisted of 14 participants (*M*_*age*_ = 59 months, age range 32–100 months). In this study, SCBP refers to sighted children of parents who are blind or have low vision. Children were included in the SCBP group if at least one of a child’s caregivers is diagnosed with visual impairments. In 13 families, one parent was diagnosed with visual impairments (2 fathers, 11 mothers). In one family, both parents are blind, however, a sighted relative lives with the family in the household. The degree of visual impairment varied and is described in more detail in Table 1. However, to include participants in the sample, it must have occurred before the child’s birth.

**TABLE 1:** Detailed characteristics of the parents who participated in the study.

No light perception or light perception only	8
Profound visual impairment (ability to see big shapes or light/dark differences with no detail)	4
Severe low vision (major difficulty with daily tasks requiring sight; relying on assistive devices)	1
Low vision (difficulties with reading standard print without magnification and recognizing faces at distance)	1

In the majority of the parents, the visual impairment was caused by genetic factors (*N* = 13), including Stargardt disease, Leber congenital amaurosis, retinal dystrophy, and coloboma. In one case (*N* = 1), the cause was retinoblastoma.

The control group consisted of 28 children reared by sighted parents, who have no close relatives with visual impairments (*M*_*age*_ = 60 months, age range 31–99 months).

The inclusion criteria for both groups were no diagnosis of systemic, genetic or metabolic conditions or neurodevelopmental disorders.

### Materials

#### Theory of Mind Task Battery (ToMTB)

Polish translation of the Theory of Mind Task Battery (ToMTB), developed by Hutchins et al. (2008) was used in the study. ToMTB is a standardized assessment designed to evaluate the developmental progression of ToM abilities in children. It comprises 15 test items embedded within nine distinct tasks, each presented through brief vignettes arranged in order of increasing difficulty. The tasks vary in both content and complexity, encompassing skills ranging from the recognition of facial expressions to the comprehension of second-order false beliefs. The test items are divided into three subscales. Early Subscale captures ToM abilities that emerge in typical development between 1 and 3 years of age. Basic Subscale intended to tap those ToM abilities that emerge in the preschool years (∼ages 3.5–5.5 years). Advanced Subscale taps those ToM abilities that emerge in later childhood (∼ages 5.5–8 years). A detailed description of the tasks included in each scale of the ToM Task Battery is provided in Table 2.

**TABLE 2:** A detailed description of the tasks included in each scale of the ToM Task Battery.

Scale	Subtasks
Early ToM	Emotion recognition: happy
	Emotion recognition: sad
	Emotion recognition: angry
	Emotion recognition: scared
	Desire-Based Emotion Task
Basic ToM	Seeing-leads-to-knowing
	Line of Sight Task (2 test questions)
	An Inference of Perception-Based Action Task
	A standard change of location false belief task
Advanced ToM	Belief-based emotion question
	Reality-based emotion question
	Second-order emotion question
	Message-desire discrepant question
	Second order False Belief question

The battery is delivered in a storybook format, with each page featuring colorful illustrations accompanied by standardized narrative text. To ensure comprehension, memory control questions are included. The experimenter reads the narratives and children are asked to indicate their answer by pointing 1 out of 4 images.

According to Hutchins et al. (2008), the ToMTB demonstrates strong psychometric properties, including satisfactory internal consistency as well as test–retest reliability. It has been validated across diverse populations.

For the basic evaluation of the psychometric properties of the Polish translation, internal consistency was evaluated for the full set of 15 dichotomously scored items using the Kuder–Richardson Formula 20 (KR-20), appropriate for binary items. The value for the full scale was KR20 = 0.61. Next, item discrimination was assessed using Pearson correlations between each item and the total score. All items showed positive discrimination, ranging from 0.19 to 0.72 (see: Table 1 and Supplementary materials).

### HEI-shifting task

Set-shifting requires children to initially create a link between a stimulus and a specific response, and then adapt by shifting from this established mental framework to form a new association (see Garon et al., 2008). Shifting skills in this study were measured with the use of the Heidelberg Shift Task (HEI-shift task; Pauen and Bechtel-Kuehne, 2016). HEI-shift task is an age-adapted version of the Dimensional Change Card Sort Task (Zelazo, 2006). In this task, two shapes (yellow square and green circle) are placed in front of participants. Children are first taught to sort white circular and square cards with a black border according to shape information (six trials). Subsequently, they receive new cards of the same two shapes in yellow and green without a black border, learning to sort them by color while ignoring shape information (six trials). Next, they receive the same cards in yellow and green but with a black border and are asked to sort the same cards by shape again while ignoring color information (six trials). The experimenter provided the child with feedback on each trial. In the final phase of the task, 18 trials were presented in a fixed order. In the final phase, the cards were mixed: the set contained colorful cards with and without the black border. Children’s task was to sort them according to the rules. The total score from this task was the total number of correct responses in the final phase.

### Explicit FBT (eFBT)

In classic FBT scenarios, visual access serves as a source of knowledge (the protagonist has either seen or not seen the object’s relocation). In order to test whether the groups differ depending on the modality included in the FBT scenarios, a custom, modified FBT was designed and developed.

The stimuli in eFBT consisted of temporally-tuned 3D animated clips featuring a protagonist who observes a toy car moving to the first box on its own. This form of task has been successfully used in previous studies (Wysocka et al., 2020). It has been modified for the present study by adding a sound. There were three conditions. False Belief (FB), where in the protagonist’s absence, the toy moves to a second box (*relocation phase*, 18–24 s). When the protagonist reenters, a recorded question “Where does the child think the toy is?” is played (*outcome explicit question phase*, 29–35 s). True Belief (TB), which is similar to FB, but the protagonist witnesses the toy’s relocation - he/she leaves and reenters the scene before the toy’s relocation, forming a true belief aligned with reality. The recorded question “Where does the child think the toy is?” is played. There is also a modified TB condition. In this condition, the protagonist does not leave the room, but turns around, thus can hear, but cannot see the toy’s relocation. The recorded question is “Where does the child think the toy is?”.

In all conditions, when the toy car moves, it produces a characteristic sound of turning wheels. Before the task, children are introduced to this sound.

There were three trials per condition and they were presented in pseudo-randomized order.

In all conditions, the participants’ answers were saved automatically after they touched the screen and stored in the log files.

### Procedure

The study procedure received approval from the local ethical committee and adhered to the principles of the Declaration of Helsinki. Written informed consent was obtained from the parents of the children participating in the study. All children agreed to taking part in the experiment. Due to recruitment challenges and the nationwide distribution of families, children from the SCBP group were primarily tested in their homes (*N* = 11). All sessions were conducted in appropriately prepared conditions, typically in a separate room to ensure a comfortable and distraction-free environment for the child. A few individual participants were assessed at the laboratory (*N* = 3). Children in the control group were tested either in a local preschool or their place of residence (*N* = 16) or at the laboratory (*N* = 12).

Each testing session lasted approximately 45 min. It started with the ToM Task Battery (approximately 10–12 min), followed by the HEI-shift task (approximately 10–12 min), and the eFBT (approximately 10 min). Children received small gifts for their participation. Additionally, financial compensation was provided to the parents: approximately 50 PLN for testing conducted at the child’s residence and approximately 150 PLN for sessions conducted at the laboratory.

## Results

### ToM Task Battery

#### Frequentist analysis

To examine whether the groups differed in their performance on the ToM Task Battery, three ANCOVAs with age as covariate, since ToM development is strongly associated with age, therefore even minor differences in average age between the two groups could explain differences in ToM scores. The ANCOVAs were performed separately for each scale (Early, Basic, Advanced) and group (SCBP, control group) as fixed factors.

For the Early scale, the analysis revealed that the effect of the group was not statistically significant, *F*(1, 37) = 1.81, *p* = 0.187. However, age in months had a significant effect, *F*(1, 37) = 14.44, *p* < 0.001.

For the Basic scale, an analogous effect was observed: the effect of the group was not statistically significant, *F*(1, 37) = 0.30, *p* = 0.59. However, age in months had a significant effect on scores, *F*(1, 37) = 15.35, *p* < 0.001.

There was no significant effect of group on Advanced ToM scores, *F*(1, 37) = 0.28, *p* = 0.60. However, age in months had a significant effect, *F*(1, *) = 24.11, *p* < 0.001.

**FIGURE 1:**
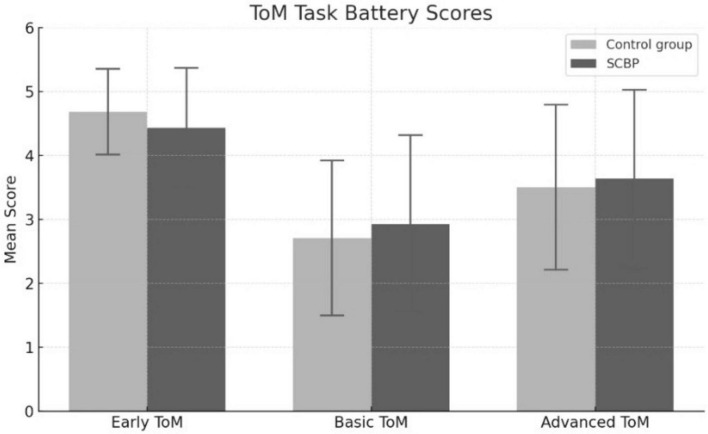
Mean score in three scales of ToM Task Battery in two groups (SCBP, control group).

Descriptive statistics are presented in Figure 1. The detailed results of ANCOVA are presented in Table 3. The results of ANOVA without including age as covariate is presented in Supplementary Table 2.

**TABLE 3:** The results of ANCOVA for each scale of ToM Task Battery, controlling for age.

Scale	Effect	df	SS	MS	F	*P*
Early ToM	Group	1	0.72	0.72	1.81	0.19
	Age (months)	1	5.77	5.77	14.44	<0.001
Basic ToM	Group	1	0.34	0.34	0.30	0.59
	Age (months)	1	17.46	17.46	15.35	<0.001
Advanced ToM	Group	1	0.32	0.32	0.28	0.60
	Age (months)	1	27.51	27.51	24.11	<0.001

SS, sum of squares; MS, mean square. All tests used Type III sum of squares.

Although the ANCOVAs revealed no significant differences between the groups, further analysis was conducted to determine whether this result reflects an actual absence of group differences or a potential lack of statistical power due to sample size limitations. To address this, a Bayesian analysis was performed. Unlike traditional null hypothesis significance testing, Bayesian methods allow for the quantification of evidence in favor of the null hypothesis, providing a more nuanced interpretation (Van de Schoot et al., 2021). This approach was used to assess the strength of evidence supporting the conclusion that SCBP and control groups do not differ meaningfully in their performance on the ToM Task Battery.

#### Bayesian analysis

Three Bayesian ANCOVAs with age as covariate were performed separately for each scale (Early, Basic, Advanced) and group (SCBP, control group) as fixed factor.

The analyses were conducted with JASP (v 0.18.1) to calculate the Bayes factor BF_01_. Since the purpose of this analysis was to support H0, we provide the BF_01_ indicator. According to the conventional interpretation, BF_01_ > 3 supports H0, while BF_01_ < .33 provides support for H1. BF01 values between 0.33 and 3 are considered inconclusive.

For the Early scale, the model including age in months alone was the best-fitting model, with a posterior model probability of *P(M|data)* = 0.596 and a Bayes factor of BF_m_ = 4.43, indicating moderate to strong evidence in favor of this model compared to all others. The BF_01_ = 0.019 shows strong evidence against the null model. The group model was poorly supported (*P(M|data)* = 0.006, *BF*_*m*_ = 0.019), suggesting strong evidence against including group as a predictor without accounting for age.

For the Basic scale, the results were analogous. The model including only age in months showed the strongest evidence, with a posterior model probability of *P(M|data)* = 0.729 and a Bayes factor of BF_m_ = 8.08, indicating strong evidence in favor of this model compared to all others. The inverse Bayes factor BF_01_ = 0.013 showed very strong evidence against the null model. The group model was poorly supported (*P(M|data)* = 0.003, *BF*_*m*_ = 0.01), and had the highest BF_01_ = 2.95, indicating moderate evidence in favor of the null over the group model.

For the Advanced scale, the results were similar: only age in months was the best fitted model, with a posterior model probability of *P(M|data)* = 0.734 and a Bayes factor of BF_m_ = 8.27, providing strong evidence in favor of this model. The BF_01_ = 0.0009 indicates very strong evidence against the null model. The group model had negligible support (*P(M|data)* = 0.00023, *BF*_*m*_ = 0.0007), and the highest BF_01_ = 3.00, providing moderate evidence for the null over the group model.

### HEI-shifting task

To examine whether the groups differed in their performance on the ToM Task Battery, ANCOVA with age as covariate and score in the HEI-shifting task as a dependent variable was performed. The analysis revealed that the effect of the group was not statistically significant, *F*(1, 34) = 0.11, *p* = 0.738. However, the covariate age in months had a significant effect on performance, *F*(1, 34) = 14.25, *p* < 0.001. The results of ANOVA without including age as covariate is presented in Supplementary Table 2.

The results were confirmed in the analogous Bayesian ANCOVA. The model including only age in months best fitted the data, with *P(M|data)* = 0.726 and BF_m_ = 7.94, indicating strong evidence in favor of this model compared to the other models. The BF_01_ = 0.019 provides very strong evidence against the null model. The group model performed poorly (*P(M|data)* = 0.005, *BF*_*m*_ = 0.014), with moderate evidence for the null model over the group-only model (*BF_01_ = 2.94*).

### eFBT

A repeated measures ANOVA was conducted to examine changes across three conditions (FB, TB, TB modified) of a within-subjects factor, group (SCBP, control) as between-subject factor, and age as covariate.

**FIGURE 2:**
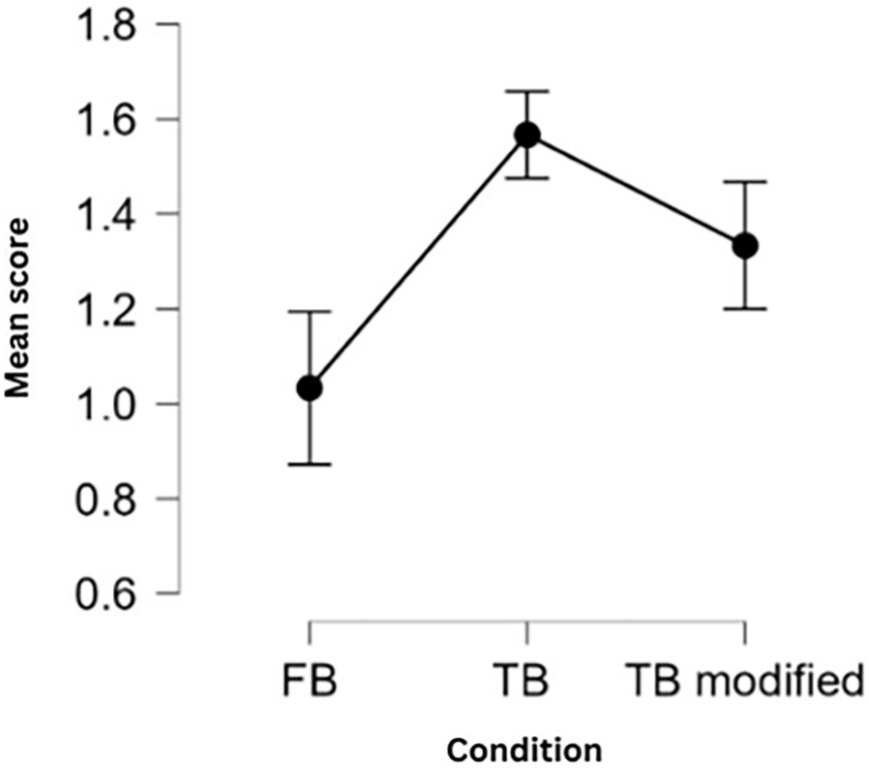
Mean scores for each eFBT condition. As the group effect was not statistically significant, results are reported for the entire sample.

There was a significant main effect of eFBT conditions *F*(2, 54) = 4.27, *p* = 0.019. *Post hoc* pairwise comparisons (Bonfferoni-Holmes corrected) revealed significant difference between FB and TB condition: *t* = −3.18, *p* = 0.007, marginally significant difference between FB and TB modified conditions: *t* = −2.25, *p* = 0.057, and no significant difference between TB and TB modified conditions: *t* = 0.93, *p* = 0.36 (Figure 2).

The interaction between eFBT conditions and age in months was marginally significant: *F*(2, 54) = 3.01, *p* = 0.058.

The interaction between eFBT conditions and group was not significant, *F*(2, 54) = 1.99, *p* = 0.14.

Analogous (condition × group × age as covariate) Bayesian rmAnova was performed. The analysis revealed that the model with eFBT conditions only was best fitted, with a posterior model probability of *P(M|data)* = 0.286 and a Bayes factor of *BF*_*m*_ = 3.61, providing moderate evidence compared to other models. The model including eFBT conditions and age was the second-best fitted (*P(M|data)* = 0.238, *BF*_*m*_ = 2.81), suggesting that adding age provides explanatory value, but not a substantial improvement. Least fitting model data was the group-only model (*P(M|data)* = 0.024, *BF*_*m*_ = 0.22), with the highest *BF_01_ = 12.04*, indicating strong evidence in favor of the null over this model.

## Discussion

The primary aim of this study was to examine social and cognitive development in children raised by parents with visual impairments - a group that has remained largely understudied. Grounded in theoretical accounts on ToM development and relation between ToM and EF, this research explored whether early exposure to a visually distinct social environment could enhance outcomes in the SCBP outcomes.

### Theory of mind performance

In contrast to our initial hypotheses, the results provided evidence that age was the only reliable predictor of ToM performance, with no additional benefit of a group. These findings were followed by Bayesian analyses, which provided strong evidence in favor of models including age alone, and moderate to strong evidence against the group effect. Notably, the group-only models consistently performed worse than the null models, particularly for the Basic and Advanced ToM subscales. One possible explanation is that the early benefits documented in infancy studies with SCBP, such as flexible adaptation of communicative strategies to blind versus sighted partners, increased vocalizations toward blind parents, reduced reliance on visual cues during interaction, and strong performance on early developmental measures, including visual attention and memory (e.g., Senju et al., 2013, 2015; Ganea et al., 2018) may not translate into enhancements in executive functioning and ToM at preschool age. Another possibility is that the age range of the current sample limited the likelihood of detecting group differences. Many children tested were already at an age where explicit FBTs are typically mastered, resulting in reduced variability and potential ceiling effects. Any SCBP-specific advantages might therefore emerge only earlier in development, for example around age three, when performance on explicit ToM tasks still shows substantial individual differences. Future studies focusing on younger cohorts may help determine whether group differences are present at earlier developmental stages.

### Executive functioning

Performance on the HEI-shift task mirrored ToM findings: no significant group differences were found, while age remained a significant predictor. Bayesian ANCOVA results again favored models including age only, indicating strong support for developmental improvements in EF but no added predictive value of early social environment. These findings contrast with some performance-based predictions. While prior studies have linked bilingualism to elevated EF skills (e.g., Kovács, 2009; McAlister and Peterson, 2013), the present findings do not support a similar boost in EF among SCBP. Several explanations are possible. First, the cognitive demands of communicating with a blind parent may differ in nature or intensity from those of switching between two or more languages. SCBP do develop adaptive strategies (e.g., adjusting eye gaze and vocalizations) from an early age, but these adjustments might become intuitive or routine within the family setting, without providing extra training in inhibitory control or attentional switching beyond the family environment. In contrast, bilingualism continuously challenges children to inhibit one language and switch to another, which may more powerfully engage and strengthen EF circuits (Bialystok and Viswanathan, 2009). Thus, not all forms of early multi-context experience produce the same EF benefits.

### Explicit false belief task (eFBT)

In the custom-designed eFBT, children showed significant differences between conditions, with better performance in TB compared to FB trials, and marginal effects for TB modified trials. Since such results were expected given the developmental trajectory of ToM (e.g., Wellman et al., 2001), they indicate the reliability of the task. However, there was no interaction with group, and Bayesian model comparisons again favored models excluding group, further reinforcing the idea that SCBP do not differ from their peers in belief attribution performance.

There are a few ways to interpret this lack of group difference. One possibility is that SCBP’s advantage, if it exists, might lie in more *implicit* or context-specific belief reasoning that standard tasks do not capture. For instance, SCBP might be especially attuned to others’ perceptual limitations in real-life interactions (e.g., spontaneously explaining things to someone who can’t see), but this strength may not translate into higher *explicit* false-belief test scores, which depend on general reasoning and language as much as on recognizing perceptual cues. This interpretation resonates with the idea that children can sometimes demonstrate understanding of others’ knowledge spontaneously or in naturalistic contexts before they succeed explicitly on structured tasks (Slaughter, 2015).

It is also important to consider that our findings align with a growing consensus that performance on explicit ToM tasks in early childhood is constrained by domain-general processing factors, such as attention, memory, and inhibitory control (Carlson and Moses, 2001). Since SCBP did not show enhanced EF in our study, it follows that they would not show enhanced ToM performance either, consistent with the tight coupling of EF and ToM at this age (Carlson et al., 2015; Devine and Hughes, 2014).

### Limitations and future directions

While the findings offer strong support for normative ToM and EF development in SCBP, several limitations should be acknowledged.

First, the sample size, especially in the SCBP group, was modest, which may limit statistical power, which has been addressed by applying Bayesian statistics. SCBP represent a highly specific and hard-to-recruit population, which poses a significant methodological challenge.

Second, the specific aspects of parental visual impairment (e.g., degree, functional adaptation, whether one or both parents have visual impairments) was, to some extent, heterogeneous. Future research should examine whether these differences may impact the outcomes. A question that remains to be addressed is whether and how the visual impairment of one parent versus both parents, as well as the level of visual acuity itself, may influence the development of the studied abilities. In the present study, however, such analyses were not possible due to the limited sample size.

Finally, the choice of measurement tools, while theoretically grounded and psychometrically validated, may not have been sufficiently sensitive to detect subtle or context-specific differences in social and cognitive functioning. It is possible that the tasks were not fine-grained enough to capture nuanced advantages or deficits that may exist within SCBP. This may especially be a case in a limited sample of children with varying experiences of either complete or partially restrained visual contact with a parent. Future studies could benefit from including additional, ecologically valid measures, such as real-time interactive tasks, naturalistic observations, or caregiver-child discourse analysis, which may better reflect the flexible and adaptive behaviors observed in infancy research.

## Data Availability

The raw data supporting the conclusions of this article will be made available by the authors, without undue reservation.
