# Supplementary material for: Theory of mind and executive functions in sighted children of blind parents
Source: Front Psychol. 2026 Feb 9;16:1715624. doi: 10.3389/fpsyg.2025.1715624 (PMC12926484; doi:10.3389/fpsyg.2025.1715624)
Supplement: Supplementary file 1 [file Table_1.docx]

**Table S1**.

*Item–rest correlation (R) values for individual test items of the ToM Task Battery.*

| Test item | R (item-rest) |
| --- | --- |
| 1 | .21 |
| 2 | .30 |
| 3 | .08 |
| 4 | .35 |
| 5 | .05 |
| 6 | .12 |
| 7 | .21 |
| 8 | .27 |
| 9 | .17 |
| 10 | .40 |
| 11 | .17 |
| 12 | .58 |
| 13 | .40 |
| 14 | .16 |
| 15 | .59 |

**Table S2**.

*The results of ANOVAs (without including age as a covariate).*

| Task | df | *F* | *p* |
| --- | --- | --- | --- |
| Early Scale (ToM Task Battery) | 1,40 | .99 | .32 |
| Basic scale (ToM Task Battery) | 1,40 | .27 | .60 |
| Advanced scale (ToM Task Battery) | 1,40 | .11 | .74 |
| HEI-shift Task | 1,37 | .06 | .80 |
